# Comparative Analysis of the Metabolites and Biological Activity of Cultivated and Wild *Lignosus rhinocerotis*

**DOI:** 10.1155/2022/5752575

**Published:** 2022-09-17

**Authors:** Bingmin Wu, Yange Wang, Lishan Zeng, Wengkun Li, Lin An, Jingyan Li, Ruoting Zhan, Huasheng Kang, Li Liu, Ying Lin, Guifang Zhang

**Affiliations:** ^1^School of Pharmaceutical Science, Guangzhou University of Chinese Medicine, Guangzhou, Guangdong 510006, China; ^2^Yishun Biological Technology Co. Ltd., Maoming, Guangdong 525000, China

## Abstract

In this paper, *Lignosus rhinocerotis* (Cooke) Ryvarden (*L. rhinocerotis*) cultivated in rice medium (LRR) and in sawdust medium (LRS) was harvested. Then, in terms of the LRR, LRS, and wild *L. rhinocerotis* (LRW), the total flavonoid contents, total polyphenol contents, total polysaccharide contents, and metabolites were detected; antioxidants of their aqueous extracts and anti-inflammatory of their polysaccharides were performed. In addition, the possible mechanism of the polysaccharides of *L. rhinocerotis* inhibiting lung damage was elucidated. The results showed that 32 compounds were characterized in *L. rhinocerotis*, including flavonoids, terpenoids, lignans, and steroids and there were 20 compounds in cultivated and wild *L. rhinocerotis*; LRR has the highest total polyphenol and flavonoid contents, as well as ABTS and DPPH scavenging capacity. The total polysaccharide contents and the FRAP scavenging capacity of wild *L. rhinocerotis* were higher than those of cultivated *L. rhinocerotis*. The inhibition of polysaccharides of LRW (PLRW) on LPS-induced MRC-5 damage was stronger than that of the polysaccharides from cultivated *L. rhinocerotis*. The PLRW may alleviate lung damage by inhibiting the NLRP3 pathway and thereby suppressing the inflammatory response. In summary, both cultivated and wild *L. rhinocerotis* are abundant in bioactive components and have antioxidant and anti-inflammatory activities.

## 1. Introduction

*Lignosus rhinocerotis* (Cooke) Ryvarden, an ethnic medicinal mushroom of the Polyporaceae family, falls into the division Basidiomycota of fungi. *L. rhinocerotis* is geographically distributed only in tropical rainforests of South China, Thailand, Malaysia, Indonesia, Philippines, and Papua New. It has been extensively used in South China, Malaysia, and other Southeast Asian countries with significant therapeutic effects on cough and asthma [1, 2]. The mature *L. rhinocerotis* consists of the sclerotium, stipe, and pileus. The sclerotium is the part of *L. rhinocerotis* with medicinal value. It is irregular hard mass with a diameter of 1–5 cm. The mature sclerotium is white, and it takes at least 3 months from mycelium to form sclerotium.

The cultivation methods mainly include solid culture and liquid culture. When the mycelium of *L. rhinocerotis* is cultured for one month, the mycelium is full of the medium and forms a layer of dense film on the surface. After three months of culture, mycelia begin to gather together to form a spherical sclerotium with a diameter of about 2 cm. At this time, sclerotium is small in size, light in color, and loose in texture. In about 6 months, sclerotium is dark and tight. A variety of active substances such as polysaccharide, polysaccharide-protein complex, protein, and protease can be extracted from sclerotium, which are anti-inflammation, antioxidative, antibacterial, and antivirus.

Mushrooms belonging to basidiomycetes are known for the presence of specific enzymes, called “ribotoxin-like proteins” whose prototype is Ageritin. This toxin possesses antifungal and antiviral activities and selective cytotoxicity against tumor cells [2]. Medicinal mushrooms produce a host of biochemicals with pharmacological effects on the process of growth, including ribotoxic proteins, bioactive peptides, polysaccharides, glycosides, terpenoids, alkaloids, steroids, and amino acids [3–7]. These bioactive substances are thought to have antitumor, antioxidant, anti-inflammatory, hypotensive, and hypocholesterolemic effects, as well as therapeutic effects on neurological diseases, liver system diseases, and respiratory system diseases [8–12]. In addition, the present reports have shown that the proteins and polysaccharides of *L. rhinocerotis* have antitumor, anti-inflammatory, and antioxidant effects [13–16]. However, the dramatic reduction in wild *L. rhinocerotis* has resulted in short supply and an artificial cultivation technique was implemented by Jamil et al. [17]. The phytochemical results showed that the total protein, total flavonoid, and total *β*-D glucan contents of cultivated *L. rhinocerotis* were higher than the wild. The mycelium could be harvested in artificially fertile living conditions, but the differences in the nutrients and the proportion of culture medium could account for its markedly different biomorphological properties, metabolites, and bioactivities. Few studies have been reported on the quality evaluation of cultivated *L. rhinocerotis* and whether cultivated *L. rhinocerotis* could be an alternative.

Pulmonary damage is an important part of the process of lung disease, and the overproduction of oxygen free radicals and inflammatory factors causes pulmonary damage, further leading to pulmonary fibrosis or lung cancer [18, 19]. Therefore, inhibition of the effects of oxidation and pulmonary damage can effectively prevent lung disease. *L. rhinocerotis* plays a critical role in the treatment of lung diseases. Previous studies have reported that the hot water extract, cold water extract, and methanolic extract of *L. rhinocerotis* have good antioxidant capacity [20]. Muhamad et al. [1] reported that the hot water extract of *L. rhinocerotis* improves the symptoms of an OVA-induced airway inflammation model in mice.

In this study, the sclerotium of LRR and LRS was harvested. The phytochemical contents (total polyphenols, total flavonoids, and total polysaccharides) of LRR, LRS, and LRW were determined. We conducted a comparison of the metabolite differences between cultivated and wild *L. rhinocerotis* by UPLC-TOF-MS combined with principal component analysis (PCA) and hierarchical cluster analysis (HCA). In addition, the antioxidant ability of the aqueous extracts of *L. rhinocerotis* was detected by free radical scavenging experiments and the protective effect of the polysaccharides of *L. rhinocerotis* (PLR) on pulmonary injury was examined by *in vitro* experiments.

## 2. Materials and Methods

### 2.1. Materials and Reagents

Ascorbic acid, formic acid, acetonitrile, quercetin, gallic acid, glucose, ethanol, phenol, sulfuric acid, Folin-Ciocalteu reagent, sodium acetate, aluminum chloride, sodium carbonate, potassium persulfate, methanol, 2,2-biphenyl-1-picrylhydrazino, 2,2-diazobis (3-ethyl-benzothiazole-6-sulfonic acid) diammonium salt, ferric chloride, 2,3,5-triphenyltetrazolium chloride, and dimethyl sulfoxide were purchased from Aladdin Biochemical Technology Co. Ltd. (Shanghai, China). The ELISA kits of tumor necrosis factor alpha (TNF-*α*), interleukin-1beta (IL-1*β*), interleukin 6 (IL-6), and interleukin 18 (IL-18) were purchased from MEIMIAN Industry Co. Ltd. (Jiangsu, China).

### 2.2. Collection and Cultivation of *L. rhinocerotis*

#### 2.2.1. *L. rhinocerotis* in the Wild (LRW)

Wild samples were collected from Baoting County, Hainan city, China, and identified as *Lignosus rhinocerotis* (Cooke) Ryvarden by gene sequencing technology at UW Genetics Corporation and Lec. Ying Lin. The experimental materials were stored in the laboratory of Guangzhou University of Chinese Medicine.

#### 2.2.2. *L. rhinocerotis* Cultivated in Rice Medium (LRR)

Mycelia were cultivated in rice medium (86% japonica rice, 4% rice bran, 8% glucose, 1% KH_2_PO_4_, 1% MgSO_4_, and M (water) : M (solid) = 65 : 35) at 25°C under dark conditions for 5 months, and then, 3~5 cm of sclerotium was harvested.

#### 2.2.3. *L. rhinocerotis* Cultivated in Sawdust Medium (LRS)

The mycelia were cultivated in sawdust medium (72% sawdust, 22% rice bran, 2% soybean meal, 1% gypsum, 3% glucose, and M (water) : M (solid) = 65 : 35) at 25°C under dark conditions for 40 days, the medium full of mycelium was buried in the soil, and then, the sclerotium was harvested when it reached 3~5 cm in diameter.

### 2.3. Extract Preparation

The dry LRW, LRR, and LRS were powdered, and then, the powder suspended in 10 volumes of distilled water was ultrasound-assisted extracted for 1 h and filtered and the extraction operation was repeated once. The supernatants were combined, condensed and freeze dried, ground into powder, and stored at −20°C.

#### 2.3.1. Polysaccharide Separation

Four volumes of 99.7% ethanol were added to the extraction solution, mixed, and rested at 4°C for 48 hours. The precipitates were collected by centrifugation, dissolved in distilled water, freeze dried, ground into powder, and stored at −20°C.

### 2.4. Total Polysaccharide, Phenolic, and Flavonoid Contents

The sample solution was 25 mg of the dry aqueous extraction of LRW, LRR, and LRS dissolved in 1 mL of distilled water.

#### 2.4.1. Total Polysaccharide Content (TPCC)

Based on the phenol-sulfuric method by Dubois et al. [21], all sample solutions were added to 9 volumes of 99.7% ethanol, mixed, and precipitated at 4°C for 48 h. The sediment was dissolved in distilled water after centrifugation at 4000 rpm for 10 min, and 500 *μ*L of the solution was incubated with 500 *μ*L of 5% phenol (*w* : *w*) and 2.5 mL of sulfuric acid (12 M) at 90°C for 15 min. The absorbance was measured at 490 nm with Ultraviolet-visible Spectrophotometer (AoYi-A390). The total polysaccharide content was expressed as *μ*g glucose equivalents per gram dry aqueous extraction (*μ*g GE/mg of DAE).

#### 2.4.2. Total Phenolic Content (TPC)

Twenty-five microliters of the solution was incubated with 25 *μ*L of Folin-Ciocalteu reagent [22] at 25°C for 5 min in the dark and then added to 25 *μ*L 10% (*w* : *w*) sodium carbonate solution and 200 *μ*L distilled water. The absorbance was measured at 765 nm. The total phenolic content was expressed as *μ*g gallic acid equivalents per gram dry aqueous extraction (*μ*g GAE/mg of DAE).

#### 2.4.3. Total Flavonoid Content (TFC)

The method was modified by Rajurkar and Hande [23]. Eighty microliters of the solution was incubated with 80 *μ*L 2% (*w* : *w*) aluminum chloride solution and 100 *μ*L sodium acetate (50 g/L) at 25°C for 2.5 h in the dark. The absorbance was measured at 415 nm. The total flavonoid content was expressed as *μ*g quercetin equivalents per gram dry aqueous extraction (*μ*g QE/mg of DAE).

### 2.5. Profile of the Metabolites Based on UPLC-TOF-MS

Aqueous extraction was performed by an AB ExionLC UPLC system equipped with an AB Triple-TOF 6600 plus high-resolution mass spectrometer with an ACQUITY UPLC HSS T3 column (100 mm × 2.1 mm, 1.8 *μ*m). Two microliters of sample solution were separated in the column at 45°C. Formic acid water (0.1%, mobile phase A) and formic acid-acetonitrile (0.1%, mobile phase B) were applied at a flow rate of 0.35 mL/min. The mobile-phase gradient elution procedure was as follows:

Mass spectrum signal acquisition was performed in ESI+ and ESI− modes. The mass spectrometry conditions were set as follows: nebulizer gas and auxiliary gas pressure 55 psi, curtain gas pressure 55 psi, interface heater temperature 550°C, ion source temperature 550°C, and mass scan range 40~1000. Mobile phase A was the solution of aqueous containing 0.1% formic acid, and mobile phase B was the solution of acetonitrile containing 0.1% formic acid. The elution procedure (A : B (*v*/*v*) at time (min)) was as follows: (95 : 5) at 0 min, (95 : 5) at 2 min, (70 : 30) at 4 min, (50 : 50) at 8 min, (20 : 80) at 10 min, (0 : 100) at 14 min, (0 : 100) at 15 min, and (95 : 5) at 16 min, and the injection volume was 2 *μ*L. The raw data were collected by UNIFI 1.8.1. software, and data processing was performed by Progenesis Q IV2.3 software (Nonlinear Dynamics, Newcastle, UK), including baseline filtering, peak identification, integration, retention time correction, peak alignment, and normalization (precursor tolerance: 5 ppm; product tolerance: 10 ppm; product ion threshold: 5%). The compounds were characterized based on their precise mass numbers, secondary fragmentation information, and isotopic distribution in The Human Metabolome Database (HMDB) and Lipidmaps (v2.3) as well as in the METLIN database. The data were extracted, the ion peaks with missing values (0 values) > 50% were removed from the group, and the 0 values were replaced by half of the minimum values and then scored according to the results of the compound characterization. The characterizations scored below 30 points were considered inaccurate (full score 60) and deleted. It was necessary to convert the raw data, including retention time, *m*/*z*, and peak intensity, to the mass-retention time pair and peak intensity. All converted information was imported into SIMCA 14.1 (Umetrics, Sweden), and then, principal component analysis (PCA) and hierarchical cluster analysis (HCA) were performed.

### 2.6. Antioxidant Activity

#### 2.6.1. ABTS Assay

According to the method and modifying by Rajurkar and Hande [23]. Ten microliters of the sample solutions and 290 *μ*L of ABTS+ radical solution mixed with 1 mL of potassium persulfate (0.7 mg/L) and 1 mL of ABTS (4 mg/L) at 25°C for 18 h in the dark were added to 96-well plates and kept at 25°C for 15 min. The absorbance was measured at 734 nm. The results are expressed as *μ*g ascorbic acid equivalents per gram dry aqueous extraction (*μ*g AAE/mg of DAE).

#### 2.6.2. DPPH Assay

The method was modified by Pande and Chanda [24]. Forty microliters of the sample solutions and 260 *μ*L of DPPH+ radical solution (40 mg/L) were added to 96-well plates and kept at 25°C for 30 min. The absorbance was measured at 517 nm. The results were expressed as *μ*g ascorbic acid equivalents per gram dry aqueous extraction (*μ*g AAE/mg of DAE).

#### 2.6.3. FRAP Assay

Based on the phenol-sulfuric method by Rajurkar and Hande [23], the FRAP solution was mixed with 0.3 M sodium acetate solution, 10 mM TPTZ solution, and 20 mM ferric chloride solution at 10 : 1 : 1. Twenty microliters of the sample solution and 280 *μ*L of the FRAP solution were added to 96-well plates and kept at 37°C for 10 min. The absorbance was measured at 593 nm. The results were expressed as *μ*g ascorbic acid equivalents per gram dry aqueous extraction (*μ*g AAE/mg of DAE).

### 2.7. Cell Line

MRC-5, a human embryonic lung fibroblast line, was provided by Procell Life Science & Technology Co. Ltd. (Wuhan, China). Cells were cultured in complete growth medium: Minimum Essential Medium *α*(MEM *α*) + 10% fetal bovine serum (FBS) + 1% penicillin-streptomycin solution (P/S) and maintained in a humidified incubator at 37°C under an atmosphere of 5% CO_2_ and 95% air. When the cells reached confluence, PBS was used to wash the cells twice and serum-free medium was used for further cell culture prior to subsequent experiments.

### 2.8. MTT Assay

Analysis of cell viability was determined by the MTT assay. Briefly, individual wells of standard flat-bottom 96-well plates were seeded with 1 × 106 MRC-5 cells per mL and incubated for 24 h at 37°C. Subsequently, the adherent cells were treated with vehicle (0.1% DMSO), various doses of PLRR (each) (20, 40, 80, 160, and 320 *μ*g/mL), PLRS (each) (20, 40, 80, 160, and 320 *μ*g/mL), or PLRW (each) (20, 40, 80, 160, and 320 *μ*g/mL). For the model group and the experimental group, we used 1 *μ*g/mL lipopolysaccharide (LPS) to stimulate the MRC-5 cells. After 48 h of incubation, the spent media were replaced with 100 *μ*L of fresh medium with 50 *μ*L of the 5 mM MTT stock solution to each well and the cells were incubated for an additional 4 h. Afterwards, the MTT formazan products were dissolved in 150 *μ*L of dimethyl sulfoxide (DMSO) and their optical densities were measured at 490 nm using a PR 601 ELISA plate reader (QualiSystems, India).

### 2.9. The Determination of Cytokines by ELISA

The concentrations of cytokines, including IL-18 (44403, MEIMIAN, China), IL-6 (14206, MEIMIAN, China), IL-1*β* (3478, MEIMIAN, China), and TNF-*α* (920, MEIMIAN, China), were measured with ELISA kits according to the manufacturer's instructions.

### 2.10. Immunofluorescence Assay

For immunofluorescence staining, all experimental cells seeded on coverslips were fixed in cold 4% paraformaldehyde (PFA) in phosphate-buffered saline (PBS) for 15 min. Samples were permeabilized with 0.3% Triton X-100 for 15 min and blocked using 5% goat serum for 30 min. Then, the samples were probed with the antibodies against ROS (diluted 1 : 1000), NLRP3 (diluted 1 : 1000), and pro-caspase-1 (diluted 1 : 1000) and incubated overnight at 4°C. After incubation with fluorescently labeled goat anti-rabbit IgG (diluted 1 : 1000), the cells were dyed with 4′,6-diamidino-2-phenylindole (DAPI) in the dark for 5 min. Images were viewed on an Olympus FV1000 fluorescence microscope.

### 2.11. Western Blot

Rabbit anti-NLRP3 polyclonal antibody (diluted 1 : 1000) and rabbit anti-caspase-1 polyclonal antibody (diluted 1 : 1000) were used in these experiments. Rabbit anti-*β*-actin polyclonal antibody (diluted 1 : 1000) served as a loading control. As described in the literature, the proteins were separated by 10% (*w*/*v*) SDS-PAGE and transferred to polyvinyl fluoride (PVDF) membranes and incubated with primary antibodies overnight at 4°C, followed by horseradish peroxidase- (HRP-) conjugated goat anti-rabbit IgG (diluted 1 : 5000) at room temperature for 2 h. Blots were developed with enhanced chemiluminescence reagent and detected by the LAS4000 imager. Densitometric analysis of the blots was performed using Image-Pro Plus 6.0 software (Rockville, MD, USA).

### 2.12. Statistical Analysis

Statistical analysis was performed using the Statistical Package for Social Sciences (SPSS for Windows, version 17). All of the data are expressed as the mean ± standard deviation (SD). Statistical differences between groups were analyzed using one-way analysis of variance (ANOVA) followed by a post hoc least-significant difference (LSD) test. *p* < 0.05 was considered to be statistically significant.

## 3. Results

### 3.1. Proximate Analysis of the Physicochemical Content

To explore the metabolite differences between cultivated and wild *L. rhinocerotis*, the physicochemical contents of LRR, LRS, and LRW were measured as shown in Table 1. The extraction rates of LRR, LRS, and LRW were 7.89%, 5.37%, and 1.86%, respectively. The TPCC of LRW was 73.15 ± 2.00 *μ*g GE/mg of DAE, which was significantly higher than that of LRR and LRS. For TFC, LRR and LRW (4.69 ± 0.07 and 4.54 ± 0.16 *μ*g QE/mg of DAE, respectively) were higher than LRS.

### 3.2. Metabolite Analysis

**Figure 1:**
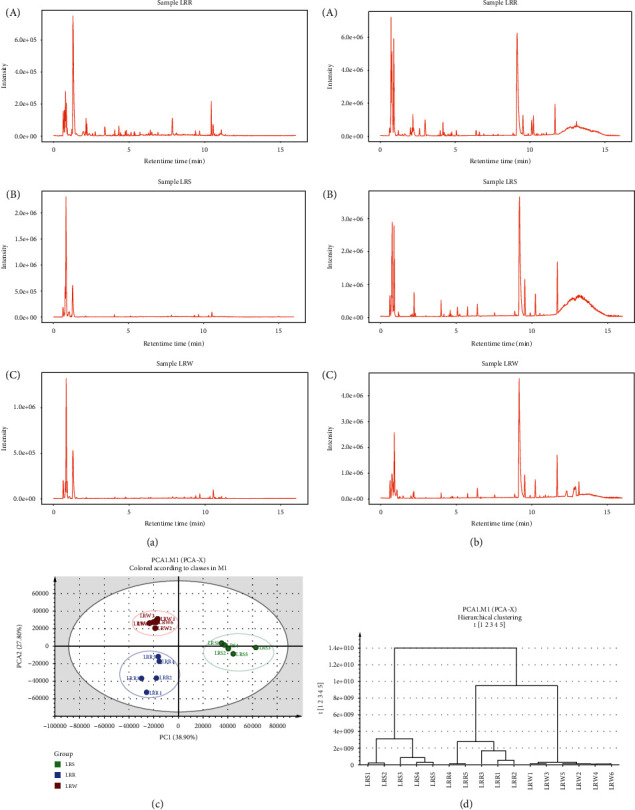
(a) Positive ion chromatogram of aqueous extracts of LRR, LRS, and LRW. (b) Negative ion chromatogram of aqueous extracts of LRR, LRS, and LRW. (c) PCA plot of LRR, LRS, and LRW. (d) HCA plot of LRR, LRS, and LRW.

The UPLC-TOF-MS technique with good efficiency and sensitivity is used to identify and characterize the biochemical compounds in food. In our study, UPLC-TOF-MS analysis led to the tentative characterization of 28, 22, and 25 compounds in LRR, LRS, and LRW, respectively. Twenty compounds were detected in LRR, LRS, and LRW, including flavonoids, terpenoids, alkaloids, and lignans. These compounds with their molecular formulas, retention times (RTs), ion modes, and errors in ppm are listed in Table 2. Figures 1(a) and 1(b) show the total ion chromatograms (both positive and negative ions) of the aqueous extracts of LRR, LRS, and LRW.

The metabolites of LRR, LRS, and LRW were characterized by untargeted analysis based on UPLC-TOF-MS. In the positive ion chromatogram, there were some of the same peaks in LRR, LRS, and LRW. However, for the negative ion chromatogram, the peaks of LRR were more intense than those of LRS and LRW. A total of 32 compounds were identified, including flavonoids (cladrastin-7-o-glucoside, isoliquiritin, angoluvarin, lupin isoflavone, rubone, tephrone, brosimacutin G, cycloartomunin, 5,2′,4′,5′-tetrahydroxy-3-(3-hydroxy-3-methylbutyl)-6′′6′′dimethylpyrano[2′′,3′′:7,8] flavone, artomunoxanthone, 3,6-dihydroxy-2-[3-methoxy-4-(sulfooxy)phenyl]-5-sulfino-3,4-dihydro-2H-1-benzopyran-7-olate, 5,7,3′-trihydroxy-4′,5′-dimethoxy-6,8-di-C-methylflavanone, [5,6,7-trihydroxy-4-oxo-2-(3,4,5-trihydroxyphenyl)-3,4-dihydro-2H-1-benzopyran-3-yl]oxidanesulfonic-acid), terpenoids (lippioside II, valtrate, armillaripin, phytuberin, and ginsenoside III), steroids (3-dehydroecdysone, 4*β*-hydroxywithanolide E, and 5-dehydroepisterol), alkaloids (cytochalasin-B and cuscohygrine), and lignans ((+)-eudesmin and dihydrodeoxy-8-epiaustdiol).

Principal component analysis (PCA) and hierarchical cluster analysis (HCA) were used to assess the differentiation of the cultivated and wild *L. rhinocerotis*. The plots of the two-dimensional PCA and HCA are presented in Figure 1. The PCA provided a map of how the LRR, LRS, and LRW were related to each other. Two components represented 66.70% of the total variation. The first component explained 38.90% of the variation, and the second component explained 27.80%. The 16 samples were divided into three clusters using HCA. LRR, LRW, and LRS were gathered into different clusters. This result indicated that there are differences in the LRR, LRS, and LRW.

### 3.3. Antioxidant Activity

The antioxidant capacity results are displayed in Table 1. For the ABTS, DPPH, and FRAP assays, LRR had the strongest ABTS and DPPH radical scavenging capacity of 24.06 ± 0.14 and 3.10 ± 0.27 *μ*g AAE/mg of DAE, respectively. The FRAP radical scavenging capacities of LRW (7.37 ± 0.18 *μ*g AAE/mg of DAE) and LRS (7.15 ± 0.20 *μ*g AAE/mg of DAE) were significantly stronger than those of LRR.

### 3.4. PLR LPS-Induced Apoptosis of MRC-5 Cells

**Figure 2:**
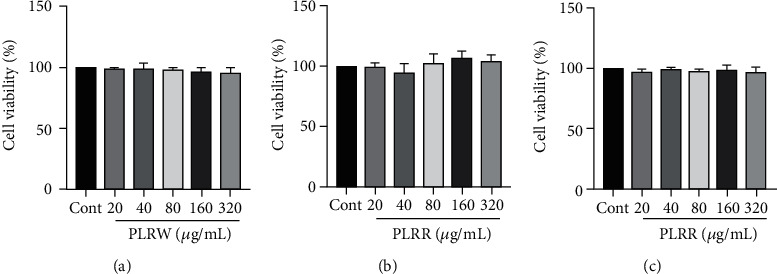
(a) The cell viability of different concentrations of PLRW on MRC-5 cell induced by LPS. (b) The cell viability of different concentrations of PLRR on MRC-5 cell induced by LPS. (c) The cell viability of different concentrations of PLRS on MRC-5 cell induced by LPS.

The viability of MRC-5 cells treated with different concentrations (20, 40, 80 160, and 320 *μ*g/mL) of PLRW, PLRR, and PLRS were examined by MTT assays. The results demonstrated that there were no differences in the cell viability of each group compared to the control (Figure 2), which suggests that 20~320 *μ*g/mL PLRR, PLRS, and PLRW have no toxic effects on MRC-5 cells.

### 3.5. PLR Reduced the Expression of Cytokines after LPS-Induced MRC-5 Injury

**Figure 3:**
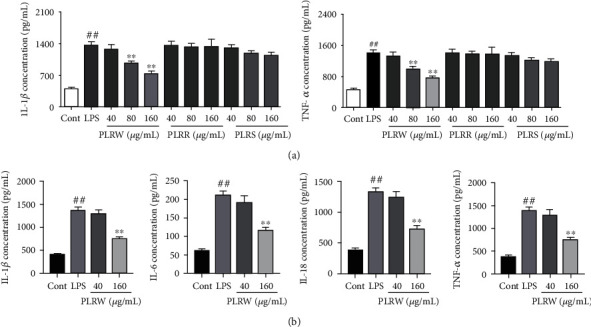
The expression of the cytokines. (a) The contents of TNF-*α* and IL-1*β* of the cell after LPS induced and pretreated with different concentrations of PLRR, PLRS, and PLRW (40~160 *μ*g/mL). (b) The contents of IL-6, IL-18, TNF-*α*, and IL-1*β* of the cell after LPS induced and pretreated with different concentrations of PLRW (40 and 160 *μ*g/mL). The results were expressed as the mean ± standard deviation (*n* = 3); compared with the LPS group, ^∗∗^*p* < 0.01 means statistically significant difference. Compared with the control group, ^##^*p* < 0.01 means statistically significant difference.

The expression of TNF-*α* and IL-1*β* were detected by enzyme-linked immunosorbent assay (ELISA). The results showed that the release of TNF-*α* and IL-1*β* in the LPS group was significantly elevated compared to that in the control group (*p* < 0.01). Compared with the LPS group, the 160 *μ*g/mL PLRW group and the 80 *μ*g/mL PLRW group had significantly reduced expression (*p* < 0.01) and the other groups did not show significant difference (Figure 3). Consequently, the levels of IL-6, IL-18, TNF-*α*, and IL-1*β* in response to the different concentrations of PLRW (40 and 160 *μ*g/mL) were detected. Likewise, their expression in the LPS group was significantly higher than that in the control group (*p* < 0.01). Compared with the LPS group, the 160 *μ*g/mL PLRW group had significantly decreased expression (*p* < 0.01), while the 40 *μ*g/mL PLRW group did not show significant difference (Figure 3).

### 3.6. PLRW Inhibited the Activation of ROS, NLRP3, and Caspase-1 after LPS-Induced MRC-5 Injury

**Figure 4:**
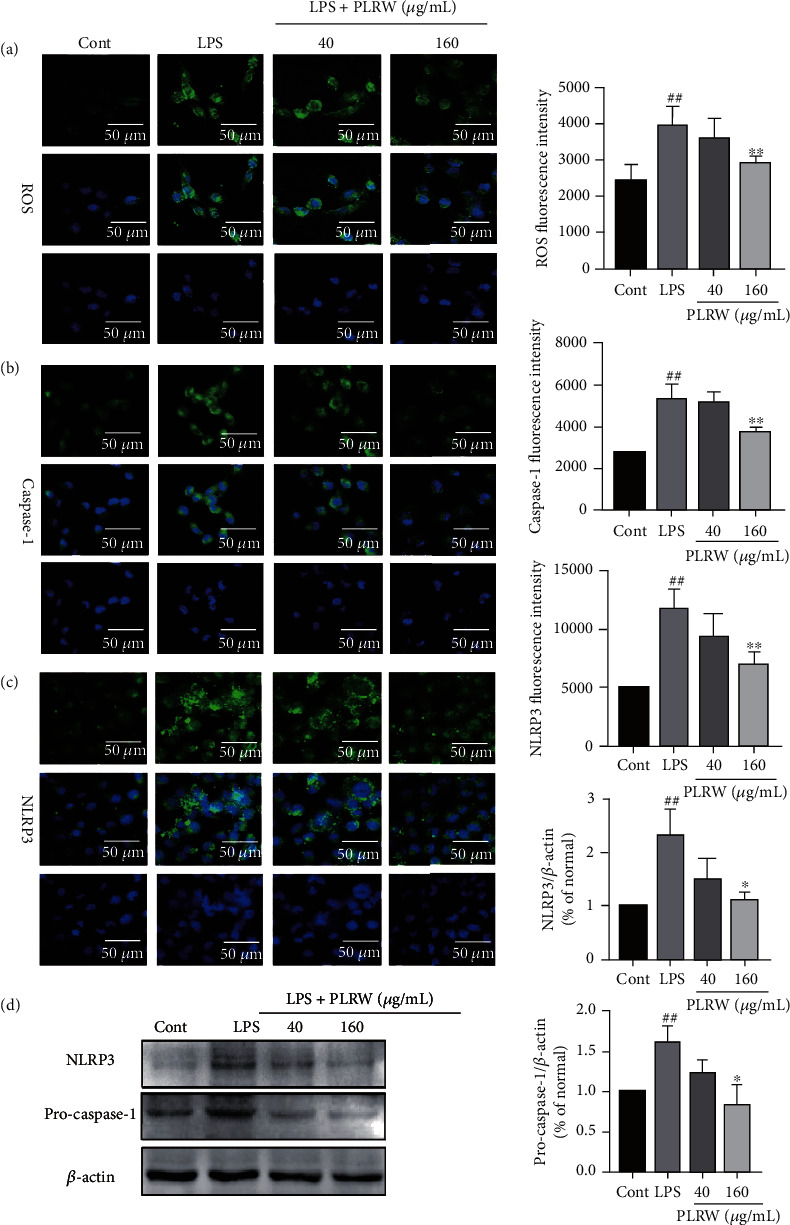
The expression of the proteins. (a) ROS expression in cells of different groups. (b) NLRP3 expression in cells of different groups. (c) Caspase-1 expression in cells of different groups. (d) The expression of NLRP3, pro-caspase-1, and *β*-actin based on Western blot. The results were expressed as the mean ± standard deviation (*n* = 3); compared with the LPS group, ^∗∗^*p* < 0.01 means statistically significant difference; ^∗^*p* < 0.05 means statistically difference. Compared with the control group, ^##^*p* < 0.01 means statistically significant difference.

Immunofluorescence assays were performed to measure the expression of ROS, NLRP3, and caspase-1. The results are shown in Figure 4. The levels of ROS, NLRP3, and caspase-1 in the LPS group were significantly increased compared to those in the control group (*p* < 0.01). Compared with the LPS group, the 160 *μ*g/mL PLRW group had significantly reduced expression (*p* < 0.01), while the 40 *μ*g/mL PLRW group did not show significant difference (*p* < 0.05).

### 3.7. PLRW Inhibited the Activation of NLRP3 and Pro-Caspase-1 after LPS-Induced MRC-5 Injury

The Western blot results showing the expression of NLRP3 and pro-caspase-1 are in Figure 4. The levels of NLRP3 and pro-caspase-1 in the LPS group were significantly increased compared to those in the control group (*p* < 0.01). Compared with the LPS group, the 160 *μ*g/mL PLRW group had significantly reduced expression (*p* < 0.01), while the 40 *μ*g/mL PLRW group did not show significant difference (*p* < 0.05).

## 4. Discussion

The bioactive compounds of medicinal mushrooms could be isolated from the mycelium, sclerotia and fruiting bodies during the growth process [25]. These biochemicals with pharmacological effects play a preventive and therapeutic role in disease. Polysaccharides are one of the therapeutic compounds of *L. rhinocerotis*, and numerous studies have shown that PLR is related to antitumor and immunomodulatory effects [26–29]. Phenolic compounds, as natural antioxidants, was able to scavenge free radicals, and the quantification of total phenolic content and total flavonoid content can be used as indicators to evaluate antioxidant capacity. Among the proximate analysis of physicochemical contents, the extraction rates of LRR (7.98%) was higher than those of LRS (5.37%) and LRW (1.86%) and the physicochemical content of cultivated LR was 4~5 times higher than wild *L. rhinocerotis*, which could be explained by the uncertain harvesting time and the harsh living conditions in the wild; conversely, the cultivated *L. rhinocerotis* was harvested under relatively fertile conditions. Per gram of dry aqueous extract, LRW had the highest polysaccharide content. LRS had the highest flavonoid and polyphenol contents. In short, both cultivated and wild *L. rhinocerotis* are abundant in bioactive compounds.

The biochemical substances of medicinal mushrooms that have inhibitory and therapeutic effects on human diseases could be identified using mass spectrometry [30]. PCA and HCA were used to detect the metabolites within *L. rhinocerotis*. In the present study, we conducted multivariate analysis for the classification and characterization of biochemicals in the *L. rhinocerotis* samples. In the PCA and HCA score plots, the LRR samples, LRS samples, and LRW samples were separated, demonstrating that there were differences among LRR, LRS, and LRW. Modern pharmacology research has revealed the bioactivities and biochemicals of medicinal mushrooms [13, 31]. At present, few studies have been reported on the metabolites of *L. rhinocerotis*. The results of the characterization of the metabolites of *L. rhinocerotis* showed that 13 flavonoids were isolated and identified, including 5 chalcones, 7 flavonoids, and 1 isoflavone. Cladrastin 7-O-glucoside (isoflavone glycosides) and lupinisoflavone M (isoflavones) were detected in LRR, isoliquiritin (flavonoid) was detected in LRR and LRS, and other flavonoids were detected in all of the samples. Lau et al. [32] used the UHPLC-ESI-MS/MS technique to analyze the metabolites in mycelium and culture broth under static liquid culture and shaking liquid culture conditions, and the main components were phenolic and fatty acids as well as lanostane triterpenoids. In this study, phenolic components, fatty acids, and lanostane triterpenes (ginsenoside III) were also detected. Moreover, 3-dehydroecdysone (insect ecdysone), 4*β*-hydroxywithanolide E (solanide), and 5-dehydroepisterol (episterol) were found in all samples and the steroids were related to anti-inflammatory and antitumor activities [33]. Furthermore, (+)-eudesmin and dihydrodeoxy-8-epiaudiol, which are lignans found in LRR, LRS, and LRW, were used to suppress the growth of bacteria and inhibit the elongation of synapses [34, 35].

Most diseases are closely related to the excessive secretion of free radicals, and the excessive production of free radicals leads to oxidative damage, which exacerbates diseases and causes a series of pathologies; thus, the exploration of natural antioxidants is increasingly urgent [36]. The aqueous extracts of *L. rhinocerotis* have good antioxidant activity [37, 38]. Antioxidant experiments, such as the ferric-reducing antioxidant power assay (FRAP), ABTS radical scavenging assay, and DPPH radical scavenging assay, were used to evaluate the antioxidant activity of various extracts [39]. For the ABTS and DPPH assays, the scavenging capacities of aqueous extracts of LRW and LRS were lower than that of LRR, which revealed that LRS has a stronger antioxidant activity. In the FRAP assay, LRW had the strongest FRAP scavenging capacity (7.37 ± 0.18 *μ*g AAE/mg of DAE). This may be related to the high content of total polyphenols and flavonoids. The mechanisms of the inhibition of redox reactions by antioxidant ingredients are different, such as producing superoxide anions, facilitating metal ion chelation, and promoting free radical chain reactions.

The NLRP3 inflammatory vesicle complex is an important signaling molecule consisting of NLRP3, the junction protein ASC, and the effector pro-caspase-1, which can be activated by various pathogen-associated molecules [40]. ROS, one of the common inflammatory vesicle activators, are secreted at increased intracellular levels when stimulated by external factors, which can facilitate the separation of TRX from TXNIP and then the integration of TXNIP with NLRP3 inflammatory vesicles [41]. However, abnormal activation of the NLRP3 inflammatory vesicle complex plays an important role in lung injury. When cells are stimulated by adverse factors, ROS are activated and stimulates NLRP3 inflammatory vesicles, leading to the activation of inflammatory vesicle-mediated caspase-1 and initiates the downstream proinflammatory cytokines IL-6, IL-18, IL-1*β*, and TNF-*α* [42]. These cytokines are powerful promoters activating the cells to release more inflammatory factors, creating a cascade amplification effect, which in turn accelerates the process of tissue damage [43]. In this study, cytotoxicity and cytokine secretion were examined after LPS-induced MRC-5 cells were pretreated with PLR and PLR at a concentration of 20~320 *μ*g/mL enhanced cell activity of LPS-induced MRC-5 cells. However, PLRR and PLRS did not show significant difference in the expression of IL-1*β* and TNF-*α*, while PLRW could significantly reduce their expression. PLRW shown good activity of anti-inflammatory than PLRR and PLRS; this is might be associated with the higher polysaccharide content.

In addition, we examined the expression of related inflammatory factors (IL-6, IL-18, IL-1*β*, and TNF-*α*) and proteins related to the NLRP3/caspase-1 pathway (ROS, NLRP3, pro-caspase-1, and caspase-1) in PLRW-pretreated MRC-5 cells. Compared to LPS-induced MRC-5 cells, the levels of IL-6, IL-18, IL-1*β*, and TNF-*α* were decreased after PLRW pretreatment and the expression levels of ROS, NLRP3, pro-caspase-1, and caspase-1 were also reduced. This result suggested that PLRW may defend against lung injury by affecting the NLRP3/caspase-1 pathway.

## 5. Conclusions

In this study, the differences between cultivated and wild *L. rhinocerotis* were elucidated. Both cultivated and wild *L. rhinocerotis* are abundant in bioactive components, and they both have antioxidant activities. This is the first time that metabolomics analysis has been applied to the study of *L. rhinocerotis* quality and the LRR, LRS, and LRW can be discriminated by untargeted metabolite profiling. In the antipulmonary injury experiments, the results suggested that wild *L. rhinocerotis* was more effective than cultivated. Moreover, PLRW plays an anti-inflammatory and antiapoptotic role in LPS-induced MRC-5 injury. PLRW may defend against cell damage by activating the NLRP3/caspase-1 pathway. In summary, both cultivated and wild *L. rhinocerotis* are abundant in bioactive components and have antioxidant and anti-inflammatory activities.

## Figures and Tables

**Table 1:** Proximate analysis of the physicochemical content and antioxidant activity.

Methods	LRR	LRS	LRW
TPCC (*μ*g GE/mg of DAE)	47.80 ± 2.96^b^	48.46 ± 2.22^b^	73.15 ± 2.00^a^
TPC (*μ*g GAE/mg of DAE)	4.91 ± 0.06^a^	4.89 ± 0.13^a^	4.85 ± 0.09^a^
TFC (*μ*g QE/mg of DAE)	4.69 ± 0.07^a^	2.17 ± 0.15^b^	4.54 ± 0.16^a^
ABTS (*μ*g AAE/mg of DAE)	24.06 ± 0.14^a^	20.78 ± 0.04^b^	17.01 ± 0.23^c^
DPPH (*μ*g AAE/mg of DAE)	3.10 ± 0.27^a^	2.45 ± 0.30^b^	1.03 ± 0.31^c^
FRAP (*μ*g AAE/mg of DAE)	2.59 ± 0.10^b^	7.15 ± 0.20^a^	7.37 ± 0.18^a^
Extraction rate	7.98%	5.37%	1.86%

In each row, different letters (a, b, and c) mean significant differences (*p* < 0.05).

**Table 2:** The metabolites of *L. rhinocerotis* were isolated and identified by UPLC-TOF-MS.

No.	Compounds	RT (min)	Ion mode	Molecular formula	ppm	*m*/*z*	Classification	LRR	LRS	LRW
1	Azlocillin	0.46	Pos	C_20_H_23_N_5_O_6_S	1.91	462.1450	Organonitrogen	^∗^	^∗^	^∗^
2	3-(3-Hydroxyphenyl)-2-phenyl-4-[(E)-2-phenylethenyl]-2,3-dihydro-1-benzofuran-6-ol	0.88	Pos	C_28_H_22_O_3_	−2.68	424.1896	Other	^∗^	—	—
3	Ergothioneine	0.91	Pos	C_9_H_15_N_3_O_2_S	0.32	230.0958	Organonitrogen	^∗^	^∗^	^∗^
4	Cladrastin 7-O-glucoside	1.14	Neg	C_24_H_26_O_11_	−8.16	535.1417	Flavonoids	^∗^	—	—
5	Lippioside II	1.15	Pos	C_25_H_30_O_14_	−4.05	537.1580	Terpenoids	^∗^	^∗^	^∗^
6	Isoliquiritin	1.19	Pos	C_21_H_22_O_9_	−9.28	436.1563	Flavonoids	^∗^	—	^∗^
7	Anacardic acid	1.49	Pos	C_22_H_30_O_3_	8.65	381.1856	Phenolic acids	^∗^	—	—
8	Angoluvarin	2.06	Pos	C_30_H_28_O_6_	4.98	502.2248	Flavonoids	^∗^	^∗^	^∗^
9	5-((5Z,8Z,11Z,14Z)-Heptadeca-5,8,11,14-tetraen-1-yl)resorcinol	3.13	Pos	C_23_H_32_O_2_	8.66	379.2063	Other	^∗^	—	—
10	Lupin isoflavone M	4.30	Pos	C_25_H_28_O_8_	−1.53	479.1669	Flavonoids	^∗^	—	—
11	Cytochalasin B	5.21	Pos	C_29_H_37_NO_5_	8.84	502.2606	Alkaloids	^∗^	^∗^	^∗^
12	Rubone	5.73	Pos	C_20_H_22_O_7_	−1.85	357.1325	Flavonoids	^∗^	^∗^	^∗^
13	Valtrate	5.74	Pos	C_10_H_12_O_5_	0.87	195.0653	Terpenoids	—	^∗^	^∗^
14	Ginsenoside III	6.34	Pos	C_48_H_80_O_19_	1.00	978.5641	Terpenoids	^∗^	^∗^	^∗^
15	Tephrone	6.40	Pos	C_18_H_16_O_6_	0.61	329.1021	Flavonoids	^∗^	^∗^	^∗^
16	Cycloartomunin	6.44	Neg	C_26_H_24_O_7_	−0.06	493.1503	Flavonoids	^∗^	^∗^	^∗^
17	5,2′,4′,5′-Tetrahydroxy-3-(3-hydroxy-3-methylbutyl)-6′′,6′′-dimethylpyrano[2′′,3′′:7,8]flavone	6.48	Pos	C_25_H_26_O_8_	3.27	477.1534	Flavonoids	^∗^	^∗^	^∗^
18	Artomunoxanthone	6.71	Neg	C_26_H_24_O_7_	−0.08	493.1503	Flavonoids	^∗^	^∗^	^∗^
19	Brosimacutin G	6.92	Pos	C_20_H_20_O_6_	−1.46	357.1334	Flavonoids	^∗^	^∗^	^∗^
20	5,7,3′-Trihydroxy-4′,5′-dimethoxy-6,8-di-C-methylflavanone	7.20	Pos	C_19_H_20_O_7_	−0.76	343.1173	Flavonoids	^∗^	^∗^	^∗^
21	Pibutidine	8.15	Neg	C_19_H_24_N_4_O_3_	−2.82	401.1820	Organonitrogen	—	—	^∗^
22	3-Dehydroecdysone	9.08	Neg	C_27_H_42_O_6_	2.16	507.2973	Steroids	—	—	^∗^
23	Cuscohygrine	9.14	Pos	C_13_H_24_N_2_O	2.03	225.1965	Alkaloids	^∗^	^∗^	^∗^
24	(+)-Eudesmin	9.88	Pos	C_22_H_26_O_6_	−0.73	387.1799	Lignans	^∗^	^∗^	^∗^
25	3,6-Hihydroxy-2-[3-methoxy-4-(sulfooxy)phenyl]-5-sulfino-3,4-dihydro-2H-1-benzopyran-7-olate	10.12	Neg	C_16_H_16_O_11_S_2_	−2.04	428.9946	Flavonoids	^∗^	^∗^	^∗^
26	Armillaripin	10.22	Pos	C_24_H_30_O_6_	0.45	415.2117	Terpenoids	^∗^	^∗^	^∗^
27	[5,6,7-Trihydroxy-4-oxo-2-(3,4,5-trihydroxyphenyl)-3,4-dihydro-2H-1-benzopyran-3-yl]oxidanesulfonic acid	10.40	Neg	C_15_H_12_O_12_S	−3.96	414.9960	Flavonoids	^∗^	^∗^	^∗^
28	4*β*-Hydroxywithanolide E	11.26	Pos	C_28_H_38_O_8_	−6.70	485.2500	Steroids	^∗^	—	^∗^
29	Phytuberin	11.40	Pos	C_17_H_26_O_4_	1.77	277.1803	Terpenoids	^∗^	^∗^	^∗^
30	Phytol	11.64	Pos	C_20_H_40_O	−2.06	314.3411	Other	^∗^	—	—
31	Dihydrodeoxy-8-epiaustdiol	11.68	Pos	C_12_H_14_O_4_	0.04	205.0859	Alkaloids	^∗^	^∗^	^∗^
32	5-Dehydroepisterol	15.91	Pos	C_2_8H_44_O	−2.18	379.3350	Steroids	—	^∗^	—

RT: retention time; pos: positive ion mode; neg: negative ion mode; ^∗^ indicates detected; — indicates not detected.

## Data Availability

The data used to support the findings of this study are included within the article.

## Abbreviations

*L. rhinocerotis*: *Lignosus rhinocerotis*
LRR: *Lignosus rhinocerotis* cultivated in rice medium
LRW: *Lignosus rhinocerotis* cultivated in wild
LRS: *Lignosus rhinocerotis* cultivated in sawdust medium
PLRR: The polysaccharides of *Lignosus rhinocerotis* cultivated in rice medium
PLRW: The polysaccharides of *Lignosus rhinocerotis* cultivated in wild
PLRS: The polysaccharides of *Lignosus rhinocerotis* cultivated in sawdust medium.
